# Selection and evaluation of an efficient method for the recovery of viral nucleic acids from complex biologicals

**DOI:** 10.1038/s41541-018-0067-3

**Published:** 2018-08-10

**Authors:** Sarmitha Sathiamoorthy, Rebecca J. Malott, Lucy Gisonni-Lex, Siemon H. S. Ng

**Affiliations:** 1 Microbiology & Virology Platform, Department of Analytical Research & Development North America, Sanofi Pasteur, Toronto, ON Canada; 2 Present Address: Turnstone Biologics, Ottawa, ON K1S 3V5 Canada

## Abstract

There is a need for a broad and efficient testing strategy for the detection of both known and novel viral adventitious agents in vaccines and biologicals. High-throughput sequencing (HTS) is an approach for such testing; however, an optimized testing method is one with a sample-processing pipeline that can help detect any viral adventitious agent that may be present. In this study, 11 commercial methods were assessed for efficient extraction of nucleic acids from a panel of viruses. An extraction strategy with two parallel arms, consisting of both the Invitrogen PureLink™ Virus RNA/DNA kit for total nucleic acid extraction and the Wako DNA Extractor^®^ kit with an RNase A digestion for enrichment of double-stranded nucleic acid, was selected as the strategy for the extraction of all viral nucleic acid types (ssRNA, dsRNA, and dsDNA). Downstream processes, such as double-strand DNA synthesis and whole-genome amplification (WGA), were also assessed for the retrieval of viral sequences. Double-stranded DNA synthesis yielded larger numbers of viral reads, whereas WGA exhibited a strong bias toward amplification of double-stranded DNA, including host cellular DNA. The final sample-processing strategy consisted of the dual extraction approach followed by double-stranded DNA synthesis, which yielded a viral population with increased detection of some viruses by 8600-fold. Here we describe an efficient extraction procedure to support viral adventitious agent detection in cell substrates used for biological products using HTS.

## Contaminants: identifying viral intruders in vaccine development

A performance-based assessment of extraction methods for biological contamination testing offers guidelines for ensuring vaccines are free of viral contaminants. Vaccine development involves the heavy use of biologicals, and limitations exist in the current methods to detect all potential viral contaminants. This prompted a team of scientists from Sanofi Pasteur, Canada, led by Siemon Ng, to test various extraction methods suitable for the detection of viral genetic material by high-throughput sequencing. The investigated methods, tested on samples spiked with four varied viruses, offered the potential to detect unknown viruses. The chosen sample preparation protocol involved the extraction of viral genetic material followed by silica-based column and precipitation followed by conversion to double-stranded DNA before sequencing. The method distilled in this paper could also be translated to other disciplines and the clinic.

## Introduction

Vaccines are among the most cost-effective public heath medical products available to date. It is estimated that for individuals born during 1994–2013, vaccination will result in net savings of $1.38 trillion (US) in total societal costs in the United States alone.^1^ With a history of safety and efficacy, vaccination is a powerful strategy to circumventing diseases. Due to the use of biological materials (e.g., cell lines, recombinant DNA, and bacterial or viral seeds) that could potentially contain microbial or viral contaminates for vaccine production, testing for adventitious agents plays a vital role in ensuring vaccine safety. Adverse effects must be avoided by ensuring the absence of contaminating infectious agents in commercially produced vaccine preparations. Guidelines for testing for adventitious agents in vaccines are provided by regulatory agencies.^2^ Viral adventitious agent testing includes in vivo assays and cell culture-based in vitro assays. These current testing methods are limited and are unable to detect a number of viral families where no suitable animal model or appropriate culturing method exists.^3^ To address these gaps in testing, target-specific nucleic acid testing (NAT) methods, such as quantitative PCR (qPCR), are used to detect the presence of viruses of interest.^4^ The use of PCR-based methods relies on a prior knowledge of the nucleic acid sequence of the viral adventitious agent for purposes of primer design, which may not always be available especially for poorly characterized or novel viruses. Work by Victoria et al.^5^ highlighted a need for an unbiased testing method for detecting viral adventitious agents. Commercially available live-attenuated vaccines were screened using high-throughput sequencing (HTS) and the presence of porcine circovirus (PCV) nucleic acid was detected in two rotavirus vaccines. PCV is not known to be infectious to humans and in the study by Victoria et al. HTS was useful in the discovery of contaminants, despite the lack of pathogenicity in humans.

HTS (also known as massively-parallel sequencing and next-generation sequencing) provides a platform for the detection of viral adventitious agents without prior knowledge of its physical and biochemical properties or its sequence content. The generation of hundreds of millions of sequencing reads facilitates the detection of low quantities of adventitious agents.^6^ The potential for high sensitivity and the ability to screen without the need for specific primers is an advantage over other methodologies available today (e.g., microarrays and PCR/qPCR). To fully utilize HTS for adventitious agent detection, an efficient sample preparation pipeline is required to ensure that the input nucleic acid provided for sequencing is representative of all the biological material in the sample. A collaborative study organized by the National Institute for Biological Standards and Control (NIBSC) using 25 different human RNA and DNA viruses illustrated that the detection of viruses by HTS is affected by different sample preparation and sequencing methods.^7^ The same NIBSC sample was also used in a study by Li et al.^8^ and highlighted that the upstream processing of samples (i.e., nucleic acid extraction, amplification, and sequencing library preparation) before sequencing by HTS can also greatly influence the sensitivity of detecting different viruses. Both studies demonstrated a need to critically assess any implemented HTS protocol for the detection of viral families representing different nucleic acid species.

Unbiased detection of low-abundance nucleic acids in different matrices, such as different cell lines and raw material, is an on-going topic of research to ensure the safety of biological products.^9–11^ This is highlighted by some of the recently published work by the Advanced Virus Detection Technologies Interest Group, which carried out a multicenter study in a cell-based matrix and spiking in a panel of five viruses.^12^ Vaccines, and in particular live or attenuated viral vaccines, can be a challenge for adventitious agent detection due to the complex matrices associated with vaccine production (e.g., cell banks, viral seeds, and viral crude harvest). Live or attenuated vaccines also require neutralization before in vitro adventitious agent testing, which may be challenging due to viral break-through and would benefit from additional methods of testing for adventitious agents other than the compendial methods that are specified in regulatory monographs.

**Fig. 1:**
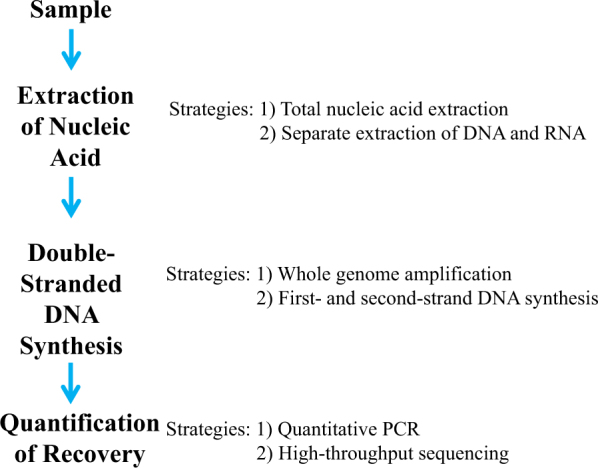
Optimization of sample-processing pipeline. The two major areas of focus for sample-processing optimization were nucleic acid extraction and double-stranded DNA synthesis. Total nucleic acid extraction and separate extraction of DNA and RNA were explored. The extracted nucleic acid was converted to single-stranded DNA before double-stranded DNA synthesis using whole-genome amplification or second-strand synthesis

Here we present an efficient end-to-end sample-processing method that was evaluated using a panel of well-characterized model viruses spiked into a HeLa cell matrix. An efficient extraction method was developed by evaluating options for key steps along the sample-processing protocol (Fig. 1). Nucleic acid extraction was evaluated by exploring total nucleic acid extraction as well as separate extraction of RNA and DNA. Extracted nucleic acid was converted to double-stranded DNA. Two strategies for double-stranded DNA synthesis were assessed: (1) whole-genome amplification (WGA) and (2) first- and second-strand synthesis with no amplification. This method is applicable to any NAT-based metaviromics protocol, including the detection of viral adventitious agents by HTS in cell substrates for biological products, such as vaccines.

## Results

### Extraction of total viral nucleic acid

Eleven commercially available extraction kits were tested for their efficient extraction of nucleic acid from HeLa cells spiked with a panel of four viruses that represent diverse biochemical and biophysical properties across different viral families: enveloped versus non-enveloped, double-stranded DNA (Epstein-Barr virus; EBV); double-stranded RNA (Reovirus 3; Reo3); single-stranded RNA (Feline leukemia virus; FeLV, and respiratory syncytial virus; RSV); and segmented genetic material (Reo3; Supplementary Information—Table 1). Extraction methods comprising silica membrane columns, magnetic beads, and nucleic acid precipitation were compared against each other. To facilitate the comparison, the QIAGEN QIAamp® MinElute® Virus Spin kit was used as the baseline, as the use of QIAGEN silica membrane-based nucleic acid extraction kits had been previously documented in viral adventitious agent testing.^8^ The data are presented as fold change in comparison to the QIAGEN QIAamp® MinElute® Virus Spin kit. A summary of the different extraction methods used and the rationale for their inclusion in this study is provided in Supplementary Information—Table 2. We found that the ClonTech Nucleobond® RNA/DNA kit allowed for separate elution of RNA and DNA but is more labor intensive, and our subsequent work demonstrated that strict separate extraction of RNA and DNA, and later combining the extraction, did not enhance detection of viruses representing either nucleic acid species. The QIAGEN QIAamp® Circulating Nucleic Acid kit accepted a larger input volume (5 mL) with ease but recovery of single-stranded RNA viruses were approximately twofold less compared to baseline. Phenol-chloroform extractions also demonstrated approximately three- and sevenfold lower recovery for FeLV and EBV, respectively.

Viral nucleic acid recovery for the four viruses was monitored using qPCR (Table 1). In general, it was found that methods using bind/elute technologies with a silica membrane or beads seem to be adept at extraction of all types of nucleic acids assessed. Methods using precipitation techniques showed evidence of poor recovery, especially against single-stranded RNA viruses. Methods that were identified as candidates for efficient viral nucleic acid recovery for different types of viral nucleic acids, such as the Invitrogen PureLink™ Virus RNA/DNA kit and the QIAGEN QIAamp® MinElute® Virus Spin kit, were assessed for repeatability (*n* = 6) and were found to be within the same order of magnitude, between extractions from the same kit, for total copy number of viral nucleic acid when controlled for the total mass (111 ng) of extracted nucleic acid used for first-strand cDNA synthesis followed by qPCR. These extraction replicates were from different samples of HeLa cells spiked with the four model viruses. For total nucleic acid recovery, Invitrogen PureLink™ Virus RNA/DNA kit reproducibly extracted higher or equal yield of viral nucleic acid, as determined by qPCR, compared to all other extraction methods tested for the efficient recovery of total nucleic acid from all four viruses.

**Table 1:** Fold change in the detection of four viruses by total nucleic acid extraction methods compared to QIAamp^®^ MinElute^®^ Virus Spin kit

Sample extraction method	Detection of EBV	Detection of FeLV	Detection of Reo3	Detection of RSV
Invitrogen PureLink™^a^	−1.55	1.68	7.89	2.31
QIAGEN Circulating Nucleic Acid^a^	−1.27	−2.50	−1.72	−1.32
Clonetech Nucleospin^®^^a^	−15.46	−62.25	−1.18	−8.00
Invitrogen Dynabeads^®^^b^	−1.02	1.22	−1.01	1.23
Perkin Elmer Chemagic™^b^	−1.49	−2.66	−5.54	−3.39
Phenol:Chloroform^c^	−27.86	−30.06	−7.52	−8.75
Acid phenol:chloroform^c^	−7.94	−3.36	9.58	1.69

Sample extraction methods were assessed for their efficient extraction of four representative viruses. Silica membrane column- (a), magnetic bead- (b), and precipitation (c)-based extractions were included

### Enriched extraction of DNA and RNA using nuclease digestion

**Fig. 2:**
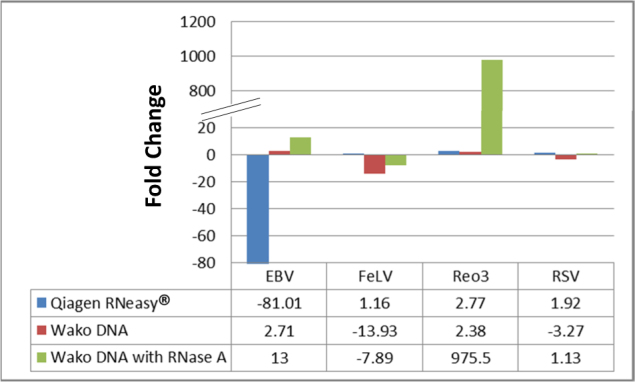
Efficiency of separate extraction of DNA and RNA when compared to total nucleic acid extraction using QIAamp® MinElute® Virus Spin kit. DNA and RNA were extracted separately and compared to the total nucleic acid extracted using the QIAamp® MinElute® Virus Spin kit, using qPCR. RNA extraction using the Qiagen RNeasy® Mini kit did not enhance the detection of the two single-stranded RNA viruses (FeLV and RSV), whereas the RNase A digestion added to the Wako DNA Extractor® kit greatly enriched for double-stranded nucleic acid (EBV and Reo3)

To explore if the efficiency of nucleic acid recovery from the matrix spiked with viruses could be improved, DNA and RNA were extracted separately with the removal of the unwanted nucleic acid using specific nuclease. RNA extraction using the QIAGEN RNeasy® Mini kit was performed with an additional DNase I digestion step after binding the nucleic acid to the column and performing an initial wash. DNA extraction using the Wako DNA Extraction® kit was performed with an RNase A digestion step during the initial Proteinase K digestion. The efficiency of nucleic acid extraction was measured by qPCR and the data are presented as fold change in comparison to the QIAGEN QIAamp® MinElute® Virus Spin kit for total nucleic acid extraction. Compared to total nucleic acid extraction using the QIAamp® MinElute® Virus Spin kit, RNA extraction using DNase I did not enhance the recovery of the two single-stranded RNA viruses, however, the RNase A digestion step enriched DNA extraction for double-stranded nucleic acids (both DNA and RNA; Fig. 2). This resulted in a large enrichment of mammalian orthoreovirus 3 (Reo3), a double-stranded RNA virus.

### Sensitivity of HTS following WGA and double-stranded DNA synthesis

We also assessed biased amplification of the extracted viral nucleic acid using WGA and/or double-stranded DNA synthesis. WGA can be applied when a low amount of starting nucleic acid is present in the sample. After the extraction of nucleic acids, all RNA was converted to double-stranded DNA prior to generating an Illumina® compatible sequencing library using the Nextera® XT DNA Library Preparation kit. To facilitate the conversion of RNA to double-stranded DNA, first-strand synthesis was performed. Efficiency of first-strand synthesis was studied using different incubation temperatures and with and without the addition of dimethyl sulfoxide, but alterations to these conditions did not increase sensitivity as measured by qPCR (data not shown). Following first-strand synthesis two techniques were examined for converting the sample to double-stranded DNA: (1) WGA; and (2) second-strand synthesis. WGA was performed using Phi29 (multiple displacement amplification—MDA), which is expected to amplify both single- and double-stranded DNA to generate double-stranded DNA.

HTS results demonstrated a large bias toward the detection of starting double-stranded DNA material when WGA was performed in comparison to double-stranded DNA synthesis (Table 2). WGA by MDA resulted in an over-representation of both background double-stranded DNA (e.g., host cell genome) and double-stranded DNA viruses, and a decrease in the sensitivity for detection of other types of viruses, such as RNA viruses.

**Table 2:** Sequencing results comparing WGA and double-stranded DNA synthesis

Virus	Double-stranded DNA synthesis		Fold change in detection
	First strand → WGA	First strand → second strand	
Total reads	342 470 258	239 327 708	
EBV	3.73% (12 770 076)	0.44% (1 049 436)	0.12
RSV	0.00038% (1287)	0.0094% (22 579)	25
FeLV	0.00030% (1032)	0.030% (71 024)	100
Reo3	0.015% (52 114)	0.31% (752 834)	21

The introduction of any bias in the viral population after WGA and/or double-stranded DNA synthesis was assessed using high-throughput sequencing. Extracted nucleic acid was converted to double-stranded DNA by first carrying out first-strand synthesis. Following first-strand synthesis the two techniques were compared for conversion to double-stranded DNA

### Selected sample preparation pipeline

**Fig. 3:**
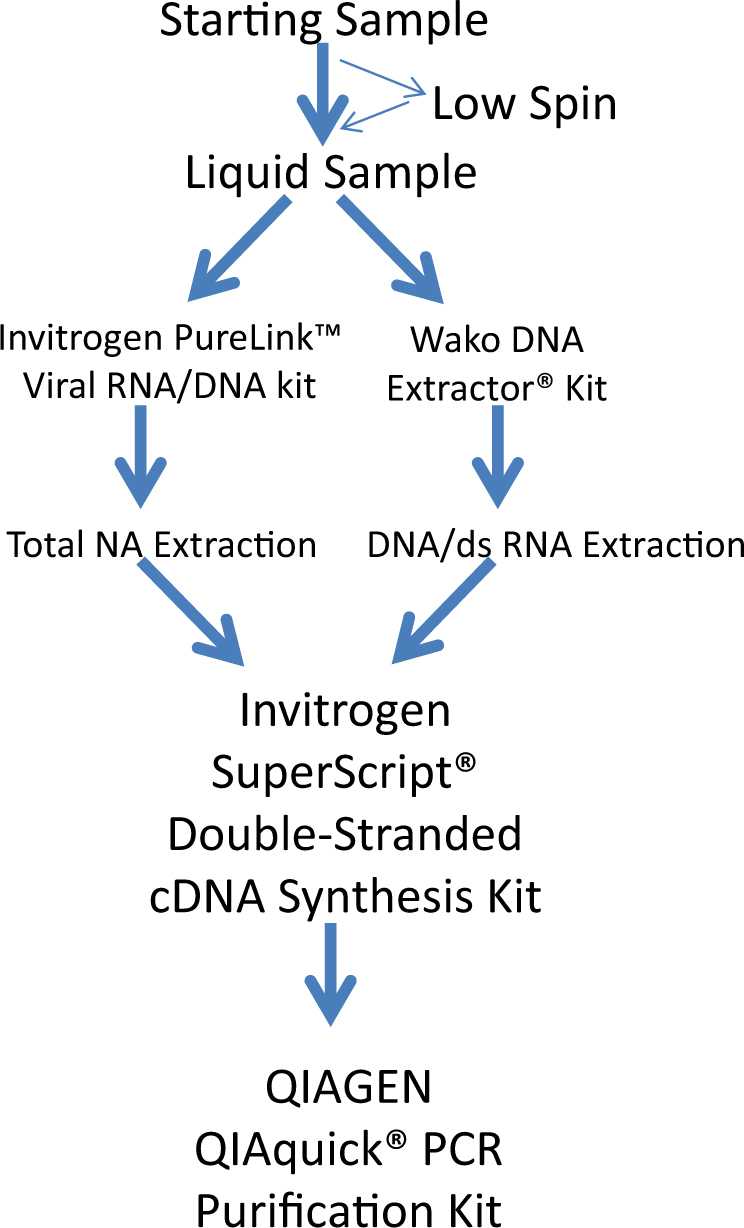
Selected sample preparation pipeline. A dual extraction strategy, using both the Invitrogen PureLink™ Virus RNA/DNA kit and the Wako DNA Extractor® kit, the latter using RNase A digestion, was selected. Extracted nucleic acid was converted to double-stranded DNA without amplification

Based on the results from the above studies a dual extraction strategy was designed, using both the Invitrogen PureLink™ Virus RNA/DNA kit (for the extraction of total nucleic acids from 200 µL of the starting sample) and the Wako DNA Extractor® kit (for the selective extraction of double-stranded nucleic acids from another 200 µL of the starting sample), with the latter including an RNase A digestion (Fig. 3). The nucleic acid recovered from virus-spiked HeLa cells was subjected to double-stranded DNA synthesis without amplification, then sequencing. All replicates were from different spiked samples that were used for the entire sample-processing protocol. This process generated a good representation of all spiked viral nucleic acid, including both double-stranded and single-stranded genetic material (Table 3). All 10 segments of the Reo3 genome were recovered. This newly devised method was compared against a total nucleic acid extraction followed by WGA to generate double-stranded DNA for sequencing library preparation. The corresponding sequencing results showed a much higher sensitivity towards all RNA viruses (both single-stranded and double-stranded) when using the optimized dual extraction strategy followed by double-stranded DNA synthesis (Table 3). Although the number of reads for double-stranded DNA virus was less when compared to the use of WGA, the total number of reads for the double-stranded DNA virus remained relatively high and the double-stranded DNA virus was readily detectable. The dual extraction, double-stranded DNA synthesis method also resulted in a large increase in the sensitivity of Reo3 virus detection.

**Table 3:** Comparison of the selected sample preparation procedure to extraction using the QIAamp^®^ MinElute^®^ Virus Spin kit followed by WGA

Virus	MinElute® + WGA (average of *n* = 3 ± standard deviation)	Optimized procedure (average of *n* = 4 ± standard deviation)	Fold change in detection
EBV	0.36 ± 0.15%	0.34% ± 0.10	0.94
RSV	0.0012 ± 0.0007%	0.0076% ± 0.0021	6.3
FeLV	0.0022 ± 0.0014%	0.036% ± 0.008	16
Reo3	0.000045 ± 0.000038%	0.38% ± 0.14	8400

The selected procedure, which involved the dual extraction strategy using both Invitrogen PureLink™ Virus RNA/DNA kit and the Wako DNA Extractor® kit with the RNase A digestion and the double-stranded DNA synthesis, was compared against the QIAGEN QIAamp® MinElute® Virus Spin kit followed by whole-genome amplification (WGA). Reported values are an average of at least three replicates with their standard deviation

## Discussion

Efficient nucleic acid extraction from samples that contain multiple viral types can be challenging to many areas such as environmental sampling, diagnostic virology, and adventitious agent testing of biologics, including vaccines. Here we showed that a dual extraction method provides improved sensitivity for recovering nucleic acids across a panel of viruses representing different biochemical and biophysical characteristics, each with their own challenges for recovery.

The viral panel studied includes a diverse set of viruses. EBV provided the simplest model material as it has a double-stranded DNA genome and does not require reverse transcription or second-strand synthesis. The EBV genome is also relatively large, making it potentially easier to detect by HTS. FeLV and RSV are single-stranded RNA viruses and the recovery levels of these viruses were used to assess any biases that may be generated by the extraction process or during the reverse transcription step against single-stranded RNA viruses. Reo3 was included to study the recovery of a double-stranded RNA virus. Double-stranded RNA may be relatively more difficult to denature and is potentially biased against during reverse transcription.^13^ The genome of Reo3 is in 10 segments of varying sizes (1.1–3.9 kb), making it possible to assess the recovery of a small segmented genome through the detection of all 10 segments using HTS.

Evaluation of separate extraction of DNA and RNA combined with specific nuclease digestion revealed that no enrichment of RNA viruses was observed in the RNA extraction with DNase digestion. We hypothesize that this may be due to either documented inefficiencies in DNase treatment using DNase I,^14^ or a high RNA to DNA ratio in the cell-derived background, which can be as high as 50-fold^15^ as seen in yeast cells. In a high background of RNA, a significant level of host RNA would remain even after the removal of DNA. In contrast, DNA extraction combined with an RNase A digestion did enrich for double-stranded DNA. Surprisingly, in addition to DNA enrichment, enrichment of double-stranded RNA was also observed. This is likely due to the efficient digestion of single-stranded RNA by RNase A that reduces the amount of total single-stranded RNA, including both cellular ribosomal and messenger RNA. Stable RNAs, such as rRNA and tRNA, can represent as much as 98% of total cellular RNA in bacteria.^16^ Activity of RNase A on rRNA is well studied^17^ and a reduction in rRNA background may enhance the relative amount of other nucleic acid in the extracted sample. rRNA depletion strategies have also been used to increase HTS assay sensitivity. Therefore, the same concentration of nucleic acid will proportionally contain more double-stranded RNA (e.g., Reo3) and DNA when the sample has undergone RNase A digest, with the preferential removal of single-stranded RNA, compared to the undigested sample (Supplementary Information to assess the Reads Per Kilobase Million is provide in Supplementary Information—Table 3).

A comparison of WGA, using MDA using Phi29, with double-stranded DNA synthesis (no amplification), allowed us to assess any bias due to this amplification step. Double-stranded DNA is required for sequencing library preparation, using the Nextera® XT DNA Sample Preparation kit, prior to HTS. The use of WGA for analytical metagenomics samples may potentially result in over-representation of double-stranded DNA extracted from viruses, bacteria, and background cellular DNA. Amplification by Phi29 is known to have a preference for long or circular, double-stranded DNA as substrate.^18^ To overcome this drawback, multiple experimental designs have incorporated the use of a ligation step.^19^ However, ligation of the starting nucleic acid material can lead to chimeric reads and to complications during data analysis.^20^ It was also noted that WGA results in a highly concentrated pool of DNA that required large dilutions prior to sequencing library preparation. This added sample dilution may lead to a decrease in the complexity of the population of nucleic acids being sequenced and impact sensitivity. In cases where WGA is unavoidable, pre-amplification methods can lead to biases through the amplification process and cause duplicate reads covering the same regions.^7^ In this case, the depth of coverage (the number of reads in the same region) may increase, however, the coverage (how much of the genome is sequenced) may be compromised. This can lead to biases in viral representation in the final sequencing reads and needs to be accounted for in the data analysis.

We found that a combination of nucleic acid extraction using the Invitrogen PureLink™ Virus RNA/DNA kit and the Wako DNA Extractor® kit supplemented with an RNase A treatment step results in the best recovery of the four viruses tested in this study. To ensure that single-stranded DNA viruses could be recovered using this strategy, PCV type 1 and minute virus of mice, both single-stranded DNA viruses, were spiked into a HeLa cell matrix and were well recovered using this extraction pipeline (data not shown).

This extraction method aids the efficient extraction of viral nucleic acids from mixed and complex samples. Here we presented its utility for viral adventitious agent testing in a cell substrate used for biological products as a representative for vaccines production. This strategy can be used when metaviromics is of interest, including other biologics, environmental testing, and clinical uses.

## Materials and methods

### Virus-spiked samples

HeLa cells were purchased from American Type Culture Collection (ATCC; catalog number CCL-2) and cells were grown and propagated using conditions described by ATCC. In all, 250 000 of the in-house propagated HeLa cells were spiked with all four viruses at the following amounts: 1.49 × 10^7^ genome copies of EBV type 1 (B95-8 strain); 1.08 × 10^9^ genome copies of FeLV (Thielen strain); 5.79 × 10^6^ genome copies of RSV subtype A (A2 strain); and 10 µL of a 1.62 × 10^7^ CCID_50_/mL stock of Reo3 (Dearing strain). EBV (catalog number 10-115-000, lot number B0116), FeLV (catalog number 10-250-000, lot number 4G0016), and RSV (catalog number 10-247-000, lot number 6J0006-PV) were purchased from Advanced Biotechnologies Inc. along with their certificate of analysis, which included genome copy number. Reo3 was purchased from Clean Cells and propagated in-house. HeLa cells that were spiked with all four viruses were stored at −80 °C until nucleic acid extraction.

### Nucleic acid extraction

Frozen spiked HeLa cells were thawed before use and centrifuged at 500 × *g* for 3 min. The supernatant was combined with nuclease-free water, if necessary, to obtain the appropriate starting volume for the method, and used for nucleic acid extraction. This ensured that all samples that were extracted contained the same absolute number of cells and viruses before extraction regardless of starting volume. Samples were extracted following manufacturer’s instructions for all of the kits tested without the addition of carrier RNA. The elution volume was standardized to 25 µL unless otherwise stated. Supplementary Information—Table 4 provides a summary of any modifications that were introduced to the manufacturer’s protocol. For the evaluation of the 11 extraction approaches for gross differences in extraction efficiency, a single sample of spiked HeLa cells was assessed. In the case of evaluating the Invitrogen PureLink™ Viral RNA/DNA kit and the QIAamp® MinElute® Virus Spin kit for reproducibility by qPCR, six replicates each, of spiked HeLa cells were extracted using the kits. All replicates used to evaluate the leading kits were from different samples of HeLa cells spiked with the four viruses. Where the double extraction method was used (Invitrogen PureLink™ Viral RNA/DNA kit and the Wako DNA Extractor® kit with RNase A digest), the initial sample was divided into two equal portions and used for extraction using each of the kits.

### qPCR assessment of virus recovery

Prior to qPCR assessment, extracted nucleic acid underwent first-strand cDNA synthesis using the SuperScript® III First-Strand Synthesis System (Invitrogen, Carlsbad, USA) following the manufacturer’s instructions, using 8 µL of extracted nucleic acid and 1 µL of random hexamer. Where the input material for the first-strand synthesis was standardized for total mass of nucleic acid, 111 ng of extracted nucleic acid was used as template material for first-strand synthesis. A volume of 5 µL of the resulting DNA was combined with 1× LightCycler® 480 SYBR Green I Master Mix (Roche, Indianapolis, USA), 500 nM of forward and reverse primers (Supplementary Information—Table 5), and PCR grade water to a final volume of 20 μL. qPCR was performed on the LightCycler® 480 Real-Time PCR System (Roche, Indianapolis, USA). The cycling protocol used for qPCR was an initial hold at 95 °C for 5 min, and 35 cycles of 95 °C for 10 s, 55 °C for 1 min, and 72 °C for 30 s. The data are represented as fold change based on C_T_ value compared to the QIAamp^®^ MinElute^®^ Virus Spin kit.

### WGA and double-stranded cDNA synthesis

Nucleic acid extracted from Invitrogen PureLink™ Viral RNA/DNA kit (containing DNA and RNA) and Wako DNA Extractor® kit with RNase A (containing DNA and double-stranded RNA) were combined in a 1:1 ratio by volume and used as template for first-strand synthesis before WGA or double-stranded DNA synthesis.

For WGA, template was first reverse-transcribed using the SuperScript® III First-Strand Synthesis System (Invitrogen, Carlsbad, USA) to create cDNA (first-strand synthesis) following the manufacturer’s instructions. A volume of 2 µL of the cDNA was used in WGA using the REPLI-g® UltraFast Mini Kit (Qiagen, Valencia, CA) and the reaction was carried out using the manufacturer’s instructions.

For double-stranded DNA synthesis using the SuperScript® Double-stranded cDNA Synthesis kit (Invitrogen, Carlsbad, USA), template was processed using the manufacturer’s instructions for starting concentration of <25 µg of total RNA. Samples were then purified and concentrated using the QIAquick® PCR purification kit (Qiagen, Valencia, CA) following the manufacturer’s instructions.

### HTS on the Illumina NextSeq 500

Indexed sequencing libraries were generated using the Nextera® XT DNA Sample Preparation kit (Illumina, San Diego, USA) following the manufacturer’s protocol and were mixed together at equal concentrations. Mixed libraries were denatured and diluted for sequencing according to the manufacturer’s protocols. Library, primers (manufacturer-supplied), and sodium hypochlorite (3 mL at 0.05%) were added to the reagent cartridge according to the manufacturer’s instructions. Libraries were sequenced using the Illumina® NextSeq® 500 (Illumina, San Diego, USA), following Illumina® sequencing protocol, with paired-end reads of 151 nucleotides each.

### Sequence data analysis

Sequence data were converted from Basecalling files (Bcl) to FASTQ sequence files and imported into Geneious Pro 6.1.3. Analysis was carried out by read mapping to reference sequences for the spiked-in viruses (EBV, accession number V01555.2; FeLV, accession number NC_001940.1; RSV, accession number KF826849.1; and Reo3, accession number GCF_000924305.1). Parameters for read mapping were as follows.

Gaps were allowed to be inserted into the reads or the reference sequence when being aligned at a maximum of 5% of the read length. A maximum size of 2 nucleotides per gap was allowed. A minimum overlap of 60 nucleotides was required for a sequence to be assembled into a contig, with a minimum identity of 75% for the overlap. A minimum of 18 consecutive nucleotides (word length) is needed to match perfectly for a match to be reported between two sequences. The index word length was set to 13 nucleotides. The threshold for mismatches was set at a maximum of 5% of single-base mismatches per read expressed as a percent of the read length. Maximum ambiguity was set at 16. Ambiguous stretches count as mismatches. In the case of multiple best matches, the read was randomly matched to one of the reference sequences.

### Data availability

Sequence data for this study are available in the NCBI SRA repository under SRA Accession Number: SRP145338.

## Electronic supplementary material

Supplementary Information

## Electronic supplementary material

**Supplementary Information** accompanies the paper on the *npj Vaccines* website (10.1038/s41541-018-0067-3).
