# Author Correction: Selection and evaluation of an efficient method for the recovery of viral nucleic acids from complex biologicals

**DOI:** 10.1038/s41541-018-0085-1

**Published:** 2018-09-24

**Authors:** Sarmitha Sathiamoorthy, Rebecca J. Malott, Lucy Gisonni-Lex, Siemon H. S. Ng

**Affiliations:** 1 Microbiology & Virology Platform, Department of Analytical Research & Development North America, Sanofi Pasteur, Toronto, ON Canada; 2 Present Address: Turnstone Biologics, Ottawa, ON K1S 3V5 Canada

**Correction to:** *npj Vaccines* (2018) 10.1038/s41541-018-0067-3, Published online 10 August 2018

In the original version of this article, the title was incorrectly given as ‘Selection and evaluation of an efficient method for the recovery of viral nucleic acid extraction from complex biologicals’. This has now been corrected in the PDF and HTML versions of the article.

