# Supplementary material for: Selection and evaluation of an efficient method for the recovery of viral nucleic acids from complex biologicals
Source: NPJ Vaccines. 2018 Aug 10;3:31. doi: 10.1038/s41541-018-0067-3 (PMC6086827; doi:10.1038/s41541-018-0067-3)
Supplement: Supplementary file 1 — Supplementary Information [file 41541_2018_67_MOESM1_ESM.pdf]

Supplementary Information - Table 1: Viruses Used and Their Associated Characteristics

4 viruses were selected to represent different biophysical and biochemical properties.

Virus	Strain	Nucleic Acid	Size of Genome (Kb) (accession number)	Enveloped
Epstein-Barr Virus	B95-8	Double-stranded DNA	172.3 (V01555.2)	Yes
Feline Leukemia Virus	Thielen	Single-stranded RNA (+ve; retrovirus)	8.2 (NC_001940.1)	Yes
Respiratory Syncytial Virus	A2	Single-stranded RNA (-ve)	15.2 (KF826849.1)	Yes
Reovirus 3	Dearing	Double-stranded RNA	23 - in 10 segments (GCF_000924305.1)	No

Supplementary Information - Table 2: Extraction Approaches Evaluated

11 extraction approaches were evaluated for unbiased extraction of viral nucleic acid extraction.

A rationale is provided for their inclusion in this study.

Supplier	Kit Name	Chemistry	Rationale for Inclusion in Study
QIAGEN (Toronto, Canada)	QIAamp® MinElute® Virus Spin Kit	Silica	Typically used sample preparation method
QIAGEN (Toronto, Canada)	QIAamp® Circulating Nucleic Acid	Silica	Up to 5 mL starting volume
QIAGEN (Toronto, Canada)	RNeasy® Mini Kit	Silica	RNA extraction
ClonTech Laboratories (Mountain View, USA)	NucleoBond® RNA/DNA	Silica	DNA and RNA separately eluted
ClonTech Laboratories (Mountain View, USA)	NucleoSpin® RNA Virus F	Silica	1 mL starting volume
Life Technologies (Carlsbad, USA)	PureLink™ Viral RNA/DNA Mini Kit	Silica	Lysis buffer is optimized for the lysis of viruses
Life Technologies (Carlsbad, USA)	Dynabeads® SILANE Viral NA Kit	Silica like beads	Not Silica; No membrane clogging by cell debris
Wako Chemicals (Richmond, USA)	DNA Extractor®	Precipitation	DNA extraction
Perkin Elmer (Waltham, USA)	Chemagic™ Viral DNA/RNA kit	Silica beads	No membrane clogging by cell debris
Life Technologies (Carlsbad, USA)	Phenol-Chloroform	Organic solvent	Potentially cleaner of salts and cell components; Large starting volume (Isolate DNA/RNA)
Life Technologies (Carlsbad, USA)	Acid Phenol-Chloroform (pH 5)	Organic solvent	Potentially cleaner of salts and cell components; Large starting volume (Isolate RNA)

Supplementary Information – Table 3: Sequencing results for the MinElute + WGA and Optimized Procedure replicates

Reads Per Kilobase Million (RPKM) is an indication of the bias observed between the different virus types (reported below in Parentheses). In the MinElute+WGA procedure, there is a large difference between the recovery of EBV and RSV / Reo3 even after normalizing for genome size. In our optimized procedure, the recovery of all viral types is more balanced with enhancement for Reo3 and RSV.

Virus	MinElute + WGA		
	Replicate #1	Replicate #2	Replicate #3
Total Number of sequencing Reads	217113184	217740212	303618486
EBV (172Kb)	1266738 (33.6)	566528 (15.1)	758085 (14.5)
RSV (8.2Kb)	510 (5.07×10^{-4})	3308 (7.38×10^{-3})	5316 (1.23×10^{-2})
FeLV (15.2Kb)	994 (66.0)	5572 (25.4)	11322 (30.9)
Reo3 (23Kb)	2 (1.02×10^{-6})	90 (1.19×10^{-4})	283 (3.09×10^{-4})

Virus	Optimized Procedure			
	Replicate #1	Replicate #2	Replicate #3	Replicate #4
Total Number of sequencing Reads	239327708	306409538	173469346	283646346
EBV (172Kb)	1049436 (25.4)	1043475 (19.8)	312890 (10.5)	1154295 (23.6)
RSV (8.2Kb)	22579 (11.5)	30172 (12.0)	8575 (6.0)	17780 (7.6)
FeLV (15.2Kb)	71024 (19.5)	137028 (29.4)	75032 (28.5)	75388 (17.5)
Reo3 (23Kb)	752834 (136.7)	580200 (82.3)	783999 (196.5)	1576400 (241.6)

Supplementary Information – Table 4: Extraction Approaches Tested and Modifications to the Manufacturer's Protocol

PureLink™ Viral RNA/DNA	Starting volume: 200 µL. Final spin to completely dry the column and the elution of the nucleic acid was carried out at 16 100 × g for 1 min.
QIAamp® MinElute® Virus Spin	Starting volume: 200 µL. Intermediate spins were carried out at 6800 × g. The columns were dried for 3 min at room temperature (rt). The final elution volume was incubated on the column at rt for 5 min and elution was at 16 100 × g
Dynabeads® SILANE Viral NA	Starting volume: 200 µL. Magnetic beads were dried for 15 min on the magnetic stand, then removed from the stand and dried for a further 5 min.
QIAamp® Circulating Nucleic Acid	Starting volume: 3 mL. The protocol for 3 mL of sample was followed. Columns were dried at rt for 10 min and the final elution was at 16 100 × g.
Chemagic™ Viral DNA/RNA	Starting volume: 200 µL. Elution was carried out with intermediate agitation.
RNeasy® Mini	Starting volume: 100 µL. Sample supernatant was combined with 15 µL of Proteinase K and incubated at rt for 10 min to digest the viral capsid. An on-column DNase digest was carried out. The second RWI wash was incubated on the column for 5 min at rt and the 2 final spins were conducted at 16 100 × g. The final elution volume was 50 µL.
Wako DNA Extractor®	Scheme 1 of the manufacturer's protocol was followed with the following modifications: Sample was combined with 20 µL Proteinase K and 0.84 µL of RNase A and incubated at 60°C for 20 min. DNA pellet was first washed with 1 mL of Wash Solution A, followed by a second wash with 1 mL of Wash Solution B with 0.5 µL of the glycogen solution. The final pellet was air-dried for 5 min and dissolved in water.
NucleoSpin® RNA Virus F	Starting volume: 1 mL. 133 µL of Proteinase K was combined with 4 mL of RAV1. To remove residual Buffer RAV3, the column was placed in a clean collection tube and spun at 3000 × g for an additional 10 min. Final elution volume was with 50 µL of water pre-heated to 70°C.
NucleoBond® RNA/DNA 80	Starting volume: 100 µL. Sample was combined with 25 µL of Proteinase K and 500 µL of Buffer W1 and incubated at 60°C for 15 min. Flow through was collected after each wash with Buffer R1, Buffer R1:R2, Buffer R2. Precipitated nucleic acid was pelleted by centrifugation at 3148 × g. Final pellet was resuspended in water.
(Acid) Phenol:Chloroform Extraction	An in-house protocol was employed. 100 µL of sample supernatant was combined with 25 µL of Proteinase K and 200 µL of the lysis buffer (from the PureLink™ Viral RNA/DNA kit) and incubated at 56°C for 15 min. 300 µL of (Acid) Phenol:Chloroform was added and vortexed for 1 min. Sample was centrifuged for 5 min at rt at 16 100 × g. Aqueous phase was combined with 2-2.5 × volume of ice-cold ethanol. Mixture was incubated at -20°C overnight and centrifuged for 30 min at 20 000 × g at 4°C. Pellet was washed with cold 80% ethanol and centrifuged for 15 min at 20 000 × g at 4°C. Supernatant was removed and pellet was dried for 15 min at rt. Final pellet was dissolved in water and incubated at 50°C for 10 min.

Supplementary Information – Table 5: Virus-Specific Primers

Virus-specific primers used for qPCR assessment of virus recovery.

Virus	Gene	Accession #	Primer Sequence 5'→3'
EBV	EBNA-1	NC_007605.1	AGCGTTTCTTGAGCTTCCCT<br>TGATGCGACGATCACAGGTC
EBV	EBNA-1	NC_007605.1	CTGGGATGAGCGTTTGG<br>CTGGCGGTCTATGATGC
EBV	EBNA-1	NC_007605.1	CAGCCCAGAGAGTAGTCTCAG<br>GTGAATCTGCTCCCAGGTCT
FeLV	LTR	NC_001940.1	CCCGTGTACGAATAAACCTC<br>CCCTGAACTAGGTCTTCCT
FeLV	LTR	NC_001940.1	TACCAGCAGTCTCCAGGCT<br>ACAGAAGCGAGAGGCGTG
RSV	N	KJ627707.1	CAGGGCAAGTGATGTTACG<br>CATAAACTTCCACAACTTGTTCC
RSV	N	KJ627707.1	TGCAGGATTGTTTATGAATGC<br>CCATTTCTGCTTGCACACTA
Reo3	M3	AF174384.1	CCTATCTACTAGAGTGTCTACCC<br>ATCCTGGAGGCTCATACC
Reo3	M3	AF174384.1	AGGCTACAGATGGAGTTGAGT<br>ATGCAAGTGTGGTGTCTCAG
